# Ultrasound Tomography Evaluation of Breast Density: A Comparison With Noncontrast Magnetic Resonance Imaging

**DOI:** 10.1097/RLI.0000000000000347

**Published:** 2017-01-24

**Authors:** Elizabeth A.M. O'Flynn, Jeremie Fromageau, Araminta E. Ledger, Alessandro Messa, Ashley D'Aquino, Minouk J. Schoemaker, Maria Schmidt, Neb Duric, Anthony J. Swerdlow, Jeffrey C. Bamber

**Affiliations:** From the *Cancer Research UK Cancer Imaging Centre; †Joint Department of Physics, Institute of Cancer Research and Royal Marsden NHS Foundation Trust; ‡Royal Marsden NHS Foundation Trust; §Division of Genetics and Epidemiology, Institute of Cancer Research, London, United Kingdom; ∥Delphinus Medical Technologies, Karmanos Cancer Institute, Wayne State University, Detroit, MI; and ¶Division of Genetics and Epidemiology, and Division of Breast Cancer Research Institute of Cancer Research, London, United Kingdom.

**Keywords:** ultrasound tomography, breast density, noncontrast Dixon MRI

## Abstract

**Objectives:**

Ultrasound tomography (UST) is an emerging whole-breast 3-dimensional imaging technique that obtains quantitative tomograms of speed of sound of the entire breast. The imaged parameter is the speed of sound which is used as a surrogate measure of density at each voxel and holds promise as a method to evaluate breast density without ionizing radiation. This study evaluated the technique of UST and compared whole-breast volume averaged speed of sound (VASS) with MR percent water content from noncontrast magnetic resonance imaging (MRI).

**Materials and Methods:**

Forty-three healthy female volunteers (median age, 40 years; range, 29–59 years) underwent bilateral breast UST and MRI using a 2-point Dixon technique. Reproducibility of VASS was evaluated using Bland-Altman analysis. Volume averaged speed of sound and MR percent water were evaluated and compared using Pearson correlation coefficient.

**Results:**

The mean ± standard deviation VASS measurement was 1463 ± 29 m s^−1^ (range, 1434–1542 m s^−1^). There was high similarity between right (1464 ± 30 m s^−1^) and left (1462 ± 28 m s^−1^) breasts (*P* = 0.113) (intraclass correlation coefficient, 0.98). Mean MR percent water content was 35.7% ± 14.7% (range, 13.2%–75.3%), with small but significant differences between right and left breasts (36.3% ± 14.9% and 35.1% ± 14.7%, respectively; *P* = 0.004). There was a very strong correlation between VASS and MR percent water density (*r*^2^ = 0.96, *P* < 0.0001).

**Conclusions:**

Ultrasound tomography holds promise as a reliable and reproducible 3-dimensional technique to provide a surrogate measure of breast density and correlates strongly with MR percent water content.

Women with dense breasts have an increased risk of developing breast cancer compared with women with less dense parenchyma.^1^ There are many different imaging techniques available to evaluate breast density, each with their advantages and disadvantages. Traditionally, 2-dimensional (2D) mammographic quantification has been widely used, reflecting differences in x-ray attenuation characteristics relating to variations in breast tissue composition on radiographic film,^2^ but 2D mammographic percent density (MPD) (percentage of fibroglandular tissue to total breast tissue) is subject to error because it is calculated from a projected image of a 3-dimensional (3D) structure of the breast.^3–6^ Furthermore, mammographic evaluation in younger women is also not routinely practiced because of the risks from ionizing radiation and poor sensitivity of cancer detection in this population.

Magnetic resonance imaging (MRI) improves on this by providing a 3D volumetric evaluation of breast density without exposure to ionizing radiation. Different MR sequences and parameters permit exploitation of inherent differences in tissue relaxation times to distinguish breast parenchyma and adipose tissue. Historically, most MRI density evaluation has been conducted on T1-weighted sequences using semiautomated segmentation of fibroglandular tissue and demonstrating good correlation with MPD^7–10^ with no consensus as to whether non–fat-suppressed^7,8,11,12^ or fat-suppressed images^13,14^ are better or more accurate. More recently, the Dixon MRI technique has been proposed as a more objective measurement of density as it provides a pure percentage water content of the breast, on the assumption that the water-only and fat-only images adequately represent the distribution of fibroglandular and adipose tissue in the breast.^15,16^ The Dixon sequence collects image data at a minimum of 2 different echo times, thereby exploiting the different relaxation properties of water and fat and producing separate high resolution water-only and fat-only images from which the volumes of fat and breast parenchyma can be estimated.^17^

Ultrasound tomography (UST) is an emerging whole-breast 3D imaging technique. A UST scan is operator-independent and covers the entire volume of the breast. The patient lies prone on the UST modified table that houses a water bath in which the breast lies dependently during scanning. An ultrasound ring sensor surrounds the breast inside the water bath and moves from the chest wall to the nipple in approximately 2 minutes while acquiring sound speed images for each position of the transducer. It has been used primarily to provide a volumetric surrogate characterization of breast density by measuring the speed of sound through tissues, which varies depending on the type of tissue, but it also images the ultrasound attenuation coefficient and tissue reflectivity and has been used as a diagnostic tool for the differentiation of benign and malignant breast lesions.^18^ The main parameter measured is the volume averaged speed of sound (VASS),^19,20^ which improves on mammographic assessment by using a whole-breast average of quantitative estimates of density generated at each voxel. The average speed of sound (*c*) through human tissue is related to the density and compressibility of the tissue as *c* ∝ (*K*/*ρ*)^1/2^ where *K* is the bulk (compressional) modulus and *ρ* is the material density of the tissue through which sound waves travel. In human breast tissue, the bulk modulus is found empirically to be related to density according to *K* ∝ *ρ.*^21–23^ Combining the 2 equations allows us to factor out the dependence on compressibility, thereby not only eliminating it as a confounding factor but also establishing a direct, empirically based linear relationship between sound speed and tissue density (*c* ∝ *ρ*).

A strong correlation between VASS and MPD has already been shown in a symptomatic population (*r*^2^ = 0.7),^24^ but VASS and MR percent water content measures of breast density have not previously been compared. The stromal and epithelial tissues of the breast that cause radio-opacification on mammography and variations in MPD are also responsible for the water content measured by MRI.^25^ Furthermore, both VASS and MR percent water use volumetric acquisitions of quantities related to density at the voxel level so are likely to have less measurement error than MPD, which is 2D and dependent on image processing to segment dense tissue. Therefore, the aim of this study was to evaluate UST clinically in a study group of asymptomatic women and compare VASS with percentage water content from a 2-point Dixon MR sequence.

## MATERIALS AND METHODS

### Subjects

This prospective single-institution cohort study had local research ethics committee approval. Written informed consent was obtained from each subject. Fifty healthy female volunteers from the generations study, a cohort study of more than 110,000 women from the general population of the United Kingdom^26^ (median age, 40 years; range, 29–64 years) were scanned between September 2014 and February 2015. Women were selected on the basis that they lived as close as possible to the hospital (to minimize travel for the subjects) and to provide a range of ages and body mass indices. Invitation letters were sent to them asking if they would like to participate. Those who responded in the affirmative were then recruited to the study. All had bilateral breast UST and 47 underwent bilateral noncontrast MRI. The UST and MRI studies were analyzed independently by 2 different individuals, one for each modality and each blinded to the results of the other.

### UST Imaging Acquisition and Analysis

**FIGURE 1:**
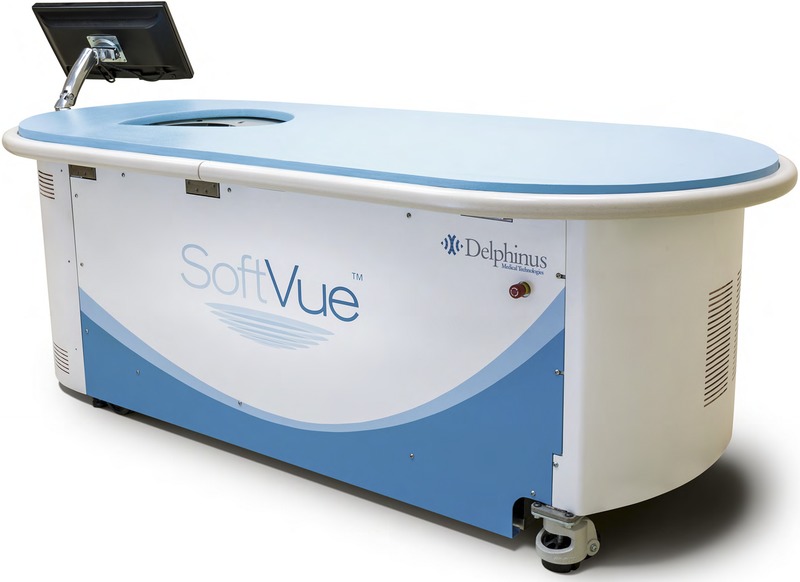
The SoftVue (Delphinus medical technologies) ultrasound tomography machine.

**FIGURE 2:**
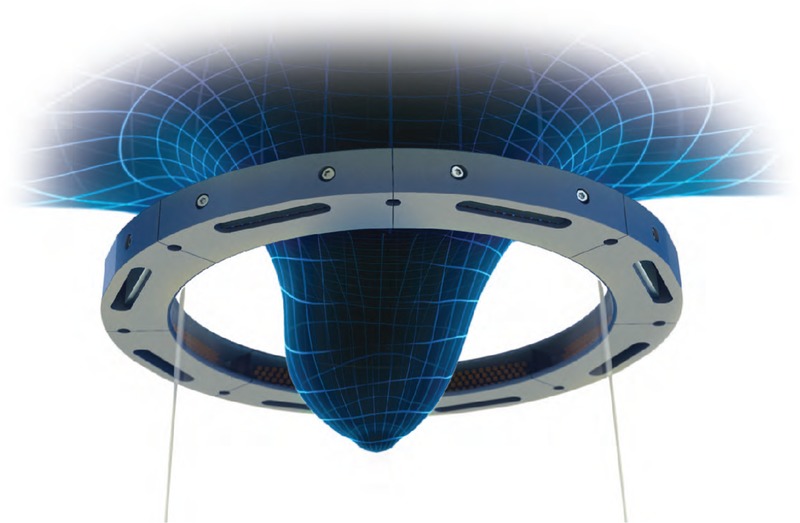
The SoftVue (Delphinus Medical Technologies) ultrasound ring array surrounds the breast and acquires images coronally moving away from the chest wall in 2.5-mm increments.

**FIGURE 3:**
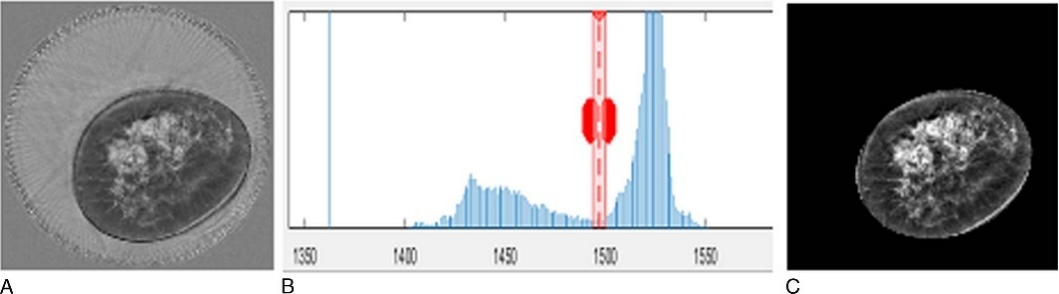
Speed of sound image (A), related histogram (B), and resulting image after applying the calculated mask (C). The contour of the mask was calculated using the semi-automated segmentation of the speed of sound images. This was done by displaying the histogram and adapting manually the threshold window (as shown by the greyed vertical band in B) between dark pixels inside the breast and the brighter pixels in the water. As shown in the B, both the threshold (represented by the position of the dotted vertical line) and the upper and lower threshold limits (represented by the outer lines) can be modified. A close contour was then interpolated using this threshold value. For subsequent images of the volume the same threshold was used automatically.

Ultrasound tomography examinations were performed on a SoftVue prototype (Delphinus Medical Technologies). The machine has been described fully in previous publications.^24,27^ The volunteer is positioned prone on the SoftVue with the breast suspended inside a warm water bath (~37°C) beneath an aperture in the table top (Fig. 1). A circumferential transducer array lies inside the water bath that contains 2048 elements within a uniform ring configuration. Initially, 1 element emits an ultrasound pulse at a central frequency of 2.5 MHz. The pulse propagates in all directions and is simultaneously recorded by the receiving elements around the ring. The sequence is then repeated by automatic 360-degree sequential excitation of the elements around the ring array and then movement of the ring array to begin the sequence again at a new distance from the chest wall, acquiring 20 to 80 coronal slices from the chest wall to the nipple at 2.5 mm increments (Fig. 2). The detector ring records sound waves, which are not reflected waves but waves that are transmitted completely through the breast. Signal arrival times are measured for many different overlapping paths through the breast. Regions of dense tissue will cause earlier arrival times, whereas fatty issues will cause later arrival times. A tomographic inversion (as in computed tomography) of these arrival times produces a sound speed map. At 2.5 MHz, attenuation of the ultrasound beam is low and there is consistent penetration of the whole breast. As the image reconstruction uses many acquisitions from different source positions, with appropriate reconstruction algorithms, the resolution of ultrasound speed tomography is much better than the resolution of a conventional ultrasound at this frequency (see, for example, the image shown later in Fig. 3).

A breast volume of interest (VOI) was obtained by manually selecting the posterior limit of the breast as the first coronal frame in which breast tissue was clearly distinct from the chest wall. This location was chosen as clearly identifiable on both UST and MRI examinations. The anterior limit of the VOI was the last frame before the nipple clear of strong reflection signal from the skin. Within the VOI, the speed of sound image stacks were summated after first defining in each slice an area of interest (AOI) using a semiautomated technique based on brightness to remove tank water and the skin signal surrounding the breast (Fig. 3). The algorithm and graphical user interface for AOI definition and summation were programmed in Matlab 2015a (Mathworks, Natick, MA). When the breast was large and very dense, the semiautomated method did not work for some slices and the AOI was defined manually. The VASS for each breast was calculated by averaging the speed of sound voxel values over the VOI.

**Formula:**
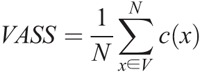


where *c*(*x*) is the voxel within the speed of sound image stacks at the position *x =* (*n*, *m*, *l*); *V* is the breast volume within the VOIs and AOIs; and *N* is the total number of voxels in *V*.

### MRI Acquisition and Analysis

Magnetic resonance imaging was performed on a 3.0 T Philips Achieva TX MRI scanner (Best, the Netherlands) using a dedicated 7-channel bilateral breast coil with the volunteer prone. An axial bilateral proton density weighted 2-point Dixon sequence was performed at high resolution (repetition time, 3.7 ms; echo time, 1.25 and 2.25 ms; reconstructed voxel size, 1.4 × 1.4 × 1.5 mm^3^; flip angle, 2 degrees) and at scan time of 61 seconds. This sequence collects image data at 2 different echo times, thereby exploiting the chemical shift differences between water and fat and produces water-only and fat-only images.^17^

For each breast, semiautomated in-house software (IDL 8.3; ITTVIS, Boulder, CO) used a combination of signal thresholding and erosion to remove the background from the in-phase Dixon image, retaining the skin.^28^ After coronal reformatting, an MR VOI for each breast was obtained according to the same rules used to derive the UST VOI; the posterior limit was defined in the same manner and an equivalent proportion of coronal slices were removed toward the nipple. To ensure a volumetric measurement of water within the breast, Dixon imaging requires a correction factor as water-only and fat-only voxels may yield different signal intensities. This correction factor was determined manually for each subject by optimizing the uniformity of the combined water and fat image and was subsequently applied to the water-only image. The water fraction (*WF*) was then calculated for every voxel within the VOI.

**Formula:**
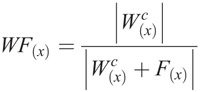


where *W*^*c*^ and *F* are the corrected water and fat signal intensities at each voxel location *x* = (*n*, *m*, *l*). The percentage of the summated voxel water fractions relative to the number of voxels within the VOI resulted in a measurement of percent water content.

### Statistical Analysis

Statistical analysis was performed using SPSS for Windows version 18 (SPSS; Chicago, IL), GraphPad Prism (GraphPad Software Inc, California), and programs written in Matlab 2015a (Mathworks, Natick, MA). Bland-Altman analysis evaluated the consistency of VASS measurements between right and left breasts. The intraclass correlation coefficient (ICC) measured the reproducibility of VASS estimates comparing values from right and left breasts, with an ICC greater than 0.75 representing good agreement.^29^ The mean VASS and MR percent water content were calculated for the whole-study group and right and left breast measurements compared using paired *t* tests. The relationship between VASS and MR percent water content was evaluated using Pearson correlation coefficient. A *P* value less than 0.05 was taken to indicate a significant difference. All reported *P* values were 2-sided.

## RESULTS

### Subjects

Of the 50 women recruited to the study, all underwent UST and 47 underwent breast MRI as 3 women were unable to tolerate the MR study, either due to claustrophobia or body habitus. MR percent water content measurements were performed on 46 of the 47 examinations as the Dixon research sequence failed to execute in 1 scan. Right breast measurements of 2 volunteers were excluded due to motion artifacts and image quality issues. A UST VOI could not be obtained for the right breast of 1 woman; thus an equivalent MRI VOI could not be defined. This left 43 paired measurements available for analysis. The median age of the study subjects was 40 years (range, 29–59 years).

### UST Imaging Results

**FIGURE 4:**
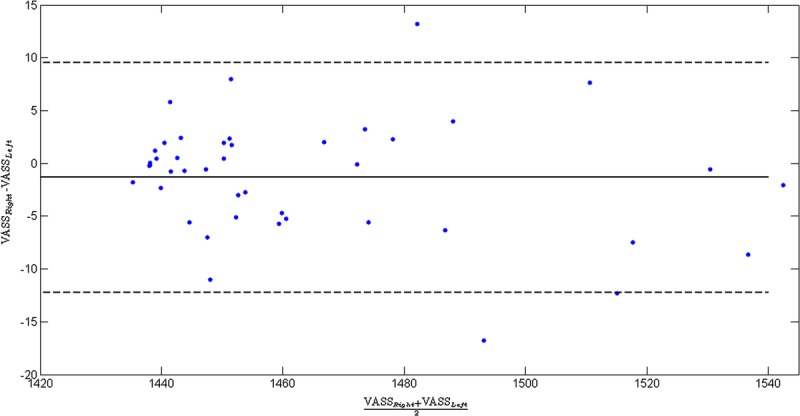
Bland-Altman plot showing the agreement of VASS result between the right and left breasts.

The mean ± standard deviation of VASS from UST for the whole cohort was 1464 ± 29 m s^−1^ (range, 1434–1542 m s^−1^). There was a very high similarity between measurements obtained from the right (1464 ± 30 m s^−1^) and left (1462 ± 28 m s^−1^) breasts (*P* = 0.113) (Table 1). The VASS from UST was found to be highly reproducible with an ICC of 0.98 (95% confidence interval, 0.97–0.99). Graphic illustration of these data in a Bland-Altman plot is shown in Figure 4.

**TABLE 1:**
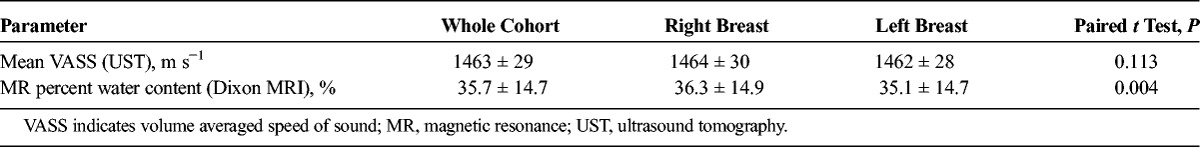
A Comparison of VASS and MR Percent Water Content in Evaluating Breast Density Between Right and Left Breasts

There was a very strong association of VASS with MR percent water content (*r*^2^ = 0.96, *P* < 0.0001). The data were modeled using the sigmoid function.

**Formula:**
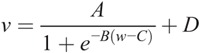


where *v* = VASS, *w* = MR percent water content, and *A*, *B*, *C*, and *D* are constants. *D* describes the low value limit of the VASS when the MR percent water content is zero and(*A* + *D*) the high value limit of the VASS when the MR percent water content is 100. For the equation of this type fitted by the method of least squares to the data, as shown in Figure 5A, these values were 1432.0 m s^−1^ and 1564.4 m s^−1^, respectively. *B* and *C* describe, respectively, the centroid of the transition and how rapidly the transition occurs, between the low and high limiting values. Representative images of women with low and high breast density are shown in Figures 6 and 7.

**FIGURE 5:**
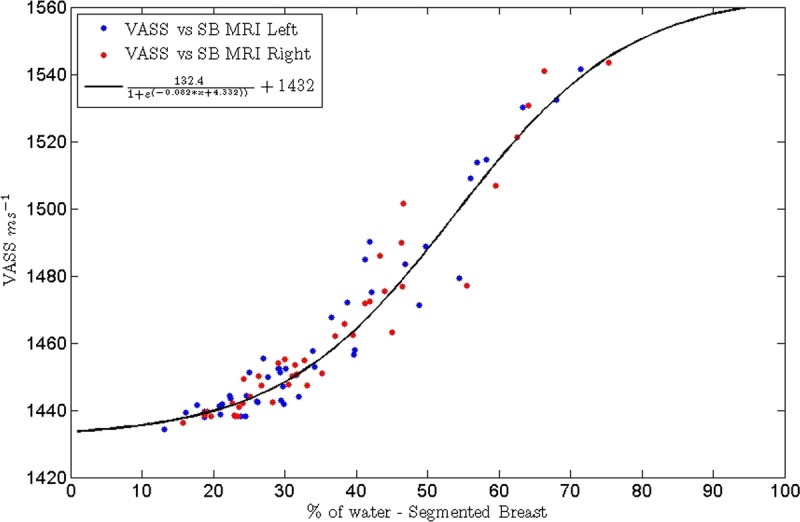
Graph illustrating the correlation between VASS and percent water content from MRI (*r*^2^ = 0.96).

**FIGURE 6:**
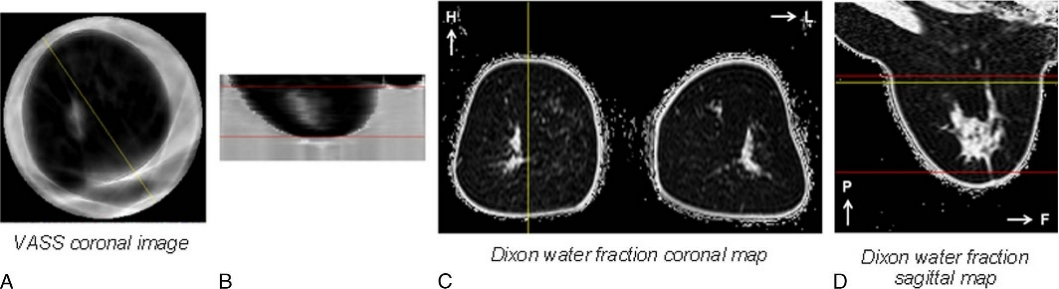
Representative UST images (A and B) and water fraction maps (C and D) of a woman with low breast density (percent water content 19.2%) - with A: Speed of Sound coronal image, B: Speed of Sound sagittal image, C: Dixon water fraction coronal map and D: Dixon water fraction sagittal map. The yellow lines indicate the relative position of depicted coronal and sagittal slices; the red lines indicate the matched coronal limits of UST and MRI VOI, and the corresponding volumes where VASS and water content were calculated.

**FIGURE 7:**
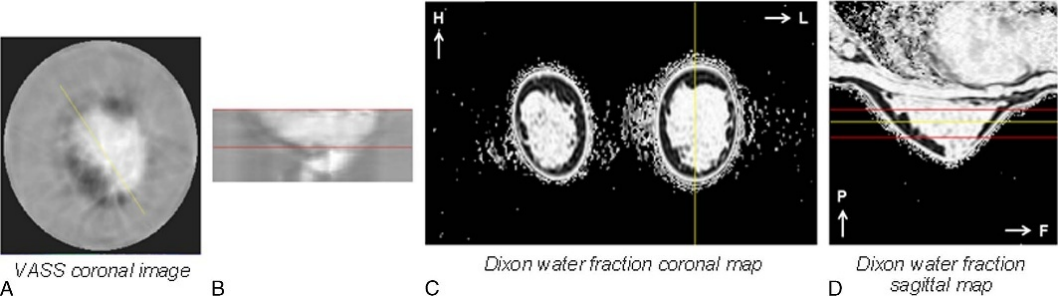
Representative UST images (A and B) and water fraction maps (C and D) of a woman with high breast density (percent water content 68.1%) - with A: Speed of Sound coronal image, B: Speed of Sound sagittal image, C: Dixon water fraction coronal map and D: Dixon water fraction sagittal map. The yellow lines indicate the relative position of depicted coronal and sagittal slices; the red lines indicate the matched coronal limits of UST and MRI VOI and the corresponding volumes where VASS and water content were calculated.

### MRI Results

Mean ± standard deviation MR percent water content for the whole-study group (43 pairs; 86 breast measures) was 35.7% ± 14.7% (range, 13.2%–75.3%). In the 43 paired measurements, there was a small but significant difference in MR percent water content between the right breast (36.3% ± 14.9%) and the left breast (35.1% ± 14.7%) (*P* = 0.004).

## DISCUSSION

This study has shown that UST can be used to provide a surrogate estimation of breast density with calculation of the VASS and that values obtained were highly comparable with those for UST in the literature.^27,30,31^ Furthermore, the limiting low value of the VASS when the MR percent water is zero, that is, a hypothetical breast that consists entirely of fat, was found to be 1432 m s^−1^, which compares favorably with a mean value of 1429 m s^−1^ (standard deviation, 25 m s^−1^; maximum, 1465 m s^−1^; minimum, 130 m s^−1^; n = 10) calculated from measurements of sound speed in fatty regions of excised human breast at body temperature, recorded over 30 years ago.^32^ The limiting high value of the VASS when the MR percent water content is 100, that is, a hypothetical breast that consists of zero fat, was found to be 1564.4 m s^−1^, which compares favorably with 1570 m s^−1^ (n = 1), a value for nonfatty human breast parenchyma measured at body temperature in excised tissue.^33^ Volume averaged speed of sound measurements were reproducible and correlated very closely with MR percent water content, indicating that VASS could be a potential alternative surrogate 3D measure of breast density.

The high association between VASS and MR percent water content can be explained in part as both are 3D density measurement techniques with quantitative voxel-by-voxel values generated for each breast in the prone position despite a more oblique patient positioning in UST relative to MRI. Also, both VASS and MR percent water content are directly measuring the proportion of adipose versus nonadipose tissue. This is different to the most widely used technique at present for evaluating breast density, which is MPD. At values below 25%, there is very little change of MR percent water density and VASS with MPD, which is similar to what has been observed previously.^24^

Breast density measurements from Dixon MRI showed significantly higher MR percent water content in the right than left breasts. This is likely a consequence of native B_0_ and B_1_ transmission-field inhomogeneity effects, which are more exaggerated at high field strength and are commonly observed in clinical breast MRI studies. Nevertheless, the positive association between VASS and MR percent water content with a sigmoidal relationship suggest that these 2 techniques provide a highly comparable quantification of the amount of fibroglandular tissue, or water-containing parenchyma in the breast. In the absence of studies directly examining the relationship between MRI-derived breast density or VASS and breast cancer risk, however, it remains to be established which modality better reflects the underlying cancer risk. Since the acquisition of these data with a Dixon research breast sequence, a product 2-point mDIXON version has since been introduced with improvements which we anticipate would increase the power of these findings; B0 demodulation and the use of a 7-peak fat model and other improvements are added to the method which were not included here.

There were several limitations to the study. First, the posterior limit of the breast in both modalities was defined as the first coronal slice excluding thoracic tissue. As a result, neither the UST nor MRI methodology measured their respective breast tissue properties further toward the chest wall and within the axilla that requires further exploration. Second, this study was performed on healthy volunteers. Several simple cysts were detected in the study group as benign incidental findings, but there were no indeterminate or malignant lesions detected and further investigation is needed to determine the influence of the presence of other benign lesions and malignancy on VASS measurements. Lastly, this study only compared breast density measurements evaluated by UST and Dixon MRI. Although the VASS from UST and MPD has been previously evaluated and shown to demonstrate a good correlation (*r*^2^ = 0.7),^24^ there is a need now for an additional prospective study comparing VASS from UST, MR-based breast density, and MPD in a matched cohort with a wide range of breast densities. Further prospective studies using UST in the diagnosis and characterization of breast lesions as well as evaluating response to neoadjuvant chemotherapy are also planned.

One of the primary potential roles for UST in the future is as a method to provide a surrogate measure of breast density in the younger woman before mammographic age, as well as women of screening age. This could potentially enable stratification of women to different screening methods based on their breast density. It is acknowledged that breast density assessment methods give a surrogate marker of risk and that there are many other different risk factors that need to be taken into account when tailoring breast screening methods. However, if UST technology provides an alternative and reliable method of assessment, it may be more appropriate in women of premammographic age to stratify for more effective breast cancer screening in the future.
